# Supplementary material for: A Murine Database of Structural Variants Identifies A Candidate Gene for a Spontaneous Murine Lymphoma Model
Source: Adv Sci (Weinh). 2026 Jan 28;13(17):e22890. doi: 10.1002/advs.202522890 (PMC13042391; doi:10.1002/advs.202522890)
Supplement: Supplementary file 1 — Supporting File 1: advs73783‐sup‐0001‐SuppMat.pdf. [file ADVS-13-e22890-s001.pdf]

# A Murine Database of Structural Variants Identifies a Candidate Gene for a Spontaneous Murine Lymphoma Model

*Wenlong Ren, Zhuoqing Fang, Egor Dolzhenko, Christopher T. Saunders, Zhuanfen Cheng, Victoria Popic, and Gary Peltz**

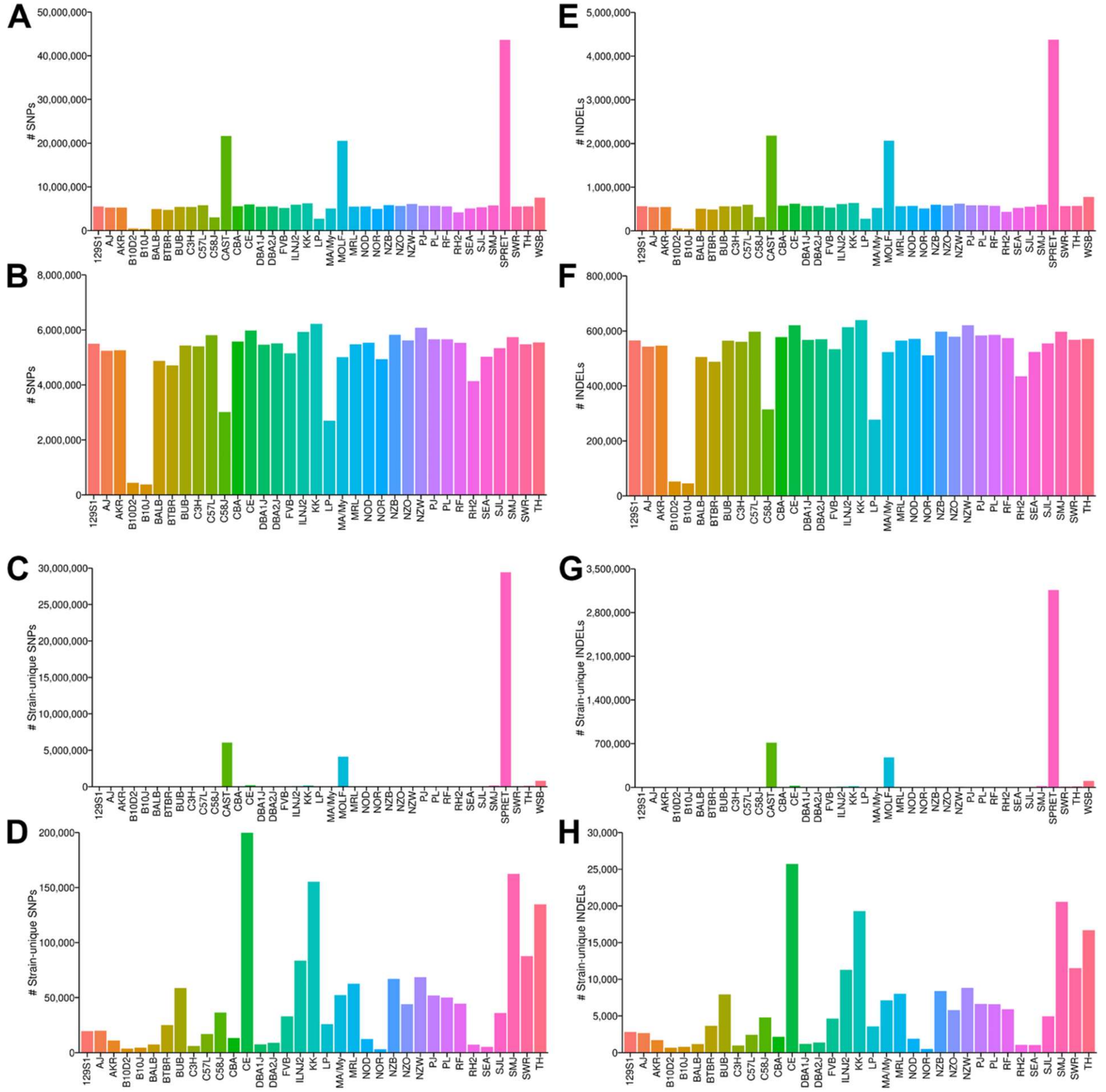


**Figure S1. SNP and INDEL discovery in all 39 strains and 35 classical inbred mouse strains.** (A-B) The number of SNP sites in (A) all 39 inbred strains or (B) in the 35 classical inbred strains against the C57BL/6J reference. (C-D) The number of sites with strain-unique SNP alleles in (C) all 39 inbred strains or (D) in the 35 classical inbred strains. Most of the strain-unique SNPs are present in the four wild derived strains (CAST, SPRET, MOLF, WSB). (E-F) The number of INDEL sites identified in (E) all 39 inbred strains or (F) in the 35 classical inbred strains against the C57BL/6J reference. (G-H) The number of sites with strain-unique INDEL alleles identified in (G) all 39 inbred strains or (H) in the 35 classical inbred strains. Most strain-unique INDELs are present in the four wild derived strains (CAST, SPRET, MOLF, WSB).

A

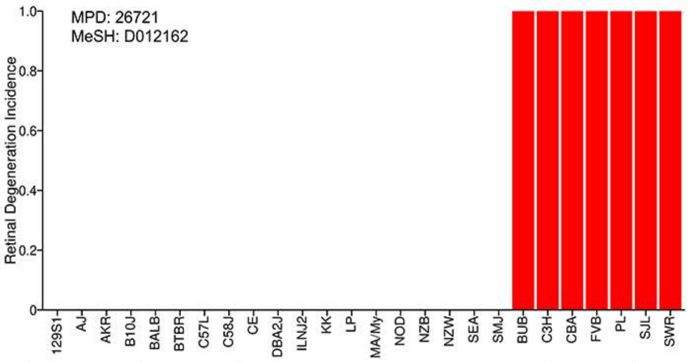


| # | Gene | SNP Flag | Haplotype | EffectSize | Pvalue | FDR | Position | LitScore | PubMed |
|---|---|---|---|---|---|---|---|---|---|
| 0 | Btbd8 | 2 | | 1.000 | 0 | 0 | 5:107582693-107585921 | 0.398 | Indirect |
| 1 | Pcgf3 | 2 | | 1.000 | 0 | 0 | 5:108609082-108616075 | 0.793 | Indirect |
| 2 | 1700028K03Rik | 2 | | 1.000 | 0 | 0 | 5:107677220-107681660 | 0.927 | Indirect |
| 3 | Pde6b | 2 | | 1.000 | 0 | 0 | 5:108537951-108543167 | 0.968 | 27203441,235932... |
| 4 | Tmem175 | 2 | | 1.000 | 0 | 0 | 5:108788563-108791304 | 0.805 | Indirect |
| 5 | Pole | 2 | | 1.000 | 0 | 0 | 5:110485520-110503524 | 0.939 | Indirect |

B

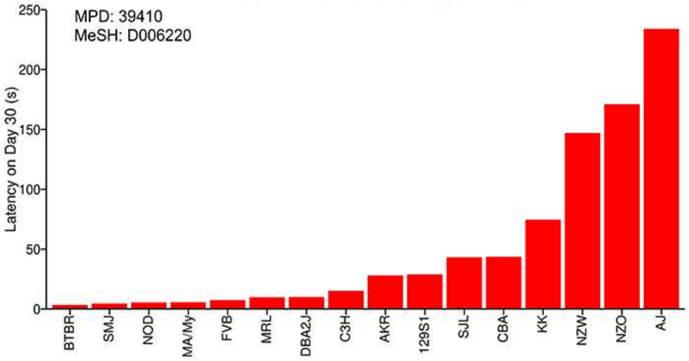


| # | Gene | SNP Flag | Haplotype | EffectSize | Pvalue | FDR | Position | LitScore | PubMed |
|---|---|---|---|---|---|---|---|---|---|
| 0 | Ctnnbl1 | 1 | | 0.955 | 4.25283e-9 | 0.0000143434 | 2:157591691-157608294 | 0.921 | Indirect |
| 1 | Vstm2l | 0 | | 0.955 | 4.25283e-9 | 0.0000143434 | 2:157759007-157762152 | 0.080 | Indirect |
| 2 | Sp8 | 1 | | 0.951 | 7.54079e-9 | 0.0000837927 | 12:118793977-118812596 | 0.940 | Indirect |
| 3 | Itgb8 | 1 | | 0.957 | 2.37158e-8 | 0.0000837927 | 12:119163326-119202835 | 0.883 | Indirect |
| 4 | Tti1 | 1 | | 0.955 | 3.04391e-8 | 0.000047382 | 2:157850645-157865836 | 0.497 | Indirect |
| 5 | Rprd1b | 0 | | 0.955 | 3.04391e-8 | 0.000047382 | 2:157893077-157926550 | 0.651 | Indirect |
| 6 | Ankrd27 | 1 | | 0.936 | 3.66389e-8 | 0.000179774 | 7:35319572-35322079 | 0.718 | Indirect |
| 7 | Pdcd5 | 1 | | 0.934 | 4.51109e-8 | 0.000179774 | 7:35345690-35345943 | 0.847 | Indirect |
| 8 | D630003M21Rik | 1 | | 0.951 | 5.05249e-8 | 0.0000681615 | 2:158011353-158037054 | 0.177 | Indirect |
| 9 | Kcns3 | 1 | | 0.904 | 6.51218e-8 | 0.0000837927 | 12:11167045-11168921 | 0.729 | Indirect |
| 10 | Abcb5 | 1 | | 0.904 | 6.51218e-8 | 0.0000837927 | 12:118822004-118834711 | 0.874 | 25647612 |
| 11 | Macc1 | 2 | | 0.904 | 6.51218e-8 | 0.0000837927 | 12:119313294-119316265 | 0.845 | Indirect |
| 12 | Slc7a10 | 1 | | 0.904 | 6.51218e-8 | 0.000179774 | 7:34889472-34889817 | 0.861 | Indirect |
| 13 | Wdr88 | 1 | | 0.904 | 6.51218e-8 | 0.000179774 | 7:34942968-34943134 | 0.000 | Indirect |
| 14 | Gpatch1 | 1 | | 0.904 | 6.51218e-8 | 0.000179774 | 7:34978249-34978731 | 0.258 | Indirect |
| 15 | Il1rapl2 | 1 | | 0.904 | 6.51218e-8 | 0.000102437 | X:136524539-136537114 | 0.598 | Indirect |

**Figure S2. Using the SNP database developed here, the AI pipeline correctly identifies the causative genetic factors for retinal degeneration (A) and resistance to the extrapyramidal effects of haloperidol (B).** The Mouse Phenome Database (MPD) has datasets examining the incidence of retinal degeneration in 26 inbred strains and the latency to move after 30 days of haloperidol treatment in 16 inbred strains, which are shown in the top graph of each panel. The AI pipeline outputs the gene symbols for that the genes it identifies as having the strongest genetic association with the phenotypic response pattern upon analysis of the published literature. The output includes the calculated p-value for the genetic association, the genetic effect size, and the chromosome with the starting and ending position of each haplotype. The color of each square within the haplotype diagram indicates the haplotype of that strain. The literature score shows the strength of the association of that gene with the measured trait, which was performed using the indicated MeSH term. Of the genes associated with retinal degeneration, *Pde6b* has a perfect genetic association score, the highest literature association score, and it is the only gene with a direct association with retinal degeneration as indicated by the indicated PubMed identification number. Of the genes associated with susceptibility to haloperidol toxicity, *Abcb5* has a strong genetic association (genetic effect size 0.9) and is the only gene with a PubMed paper that is directly linked with the trait. *Pde6b* and *Abcb5* were previously shown to contain the causative murine genetic factors for retinal degeneration and susceptibility to haloperidol toxicity, respectively.

**A**

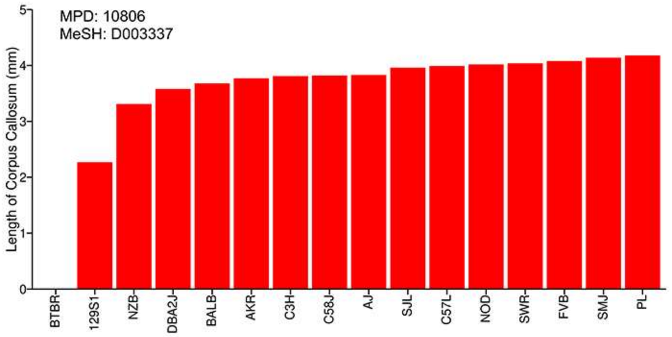


| # | Gene | SNP Flag | Haplotype | EffectSize | Pvalue | FDR | Position | LitScore | PubMed |
|---|---|---|---|---|---|---|---|---|---|
| 0 | Cd180 | 2 | | 0.785 | 0.00000211382 | 0.000659641 | 13:102850747-102859716 | 0.912 | Indirect |
| 1 | Parp10 | 2 | | 0.785 | 0.00000211382 | 0.000236118 | 15:76127135-76127135 | 0.852 | Indirect |
| 2 | Draxin | 2 | | 0.785 | 0.00000211382 | 0.000342675 | 4:148200038-148200038 | 0.832 | 19150847,23206892 |
| 3 | Hcfc1r1 | 2 | | 0.771 | 0.0000750136 | 0.00511472 | 17:23890264-23903432 | 0.871 | Indirect |
| 4 | Slc22a16 | 2 | | 0.415 | 0.00345635 | 0.0706927 | 10:40353401-40503418 | 0.961 | Indirect |
| 5 | Galntl5 | 2 | | 0.337 | 0.042536 | 0.47682 | 5:25377931-25513025 | 0.424 | Indirect |

**B**

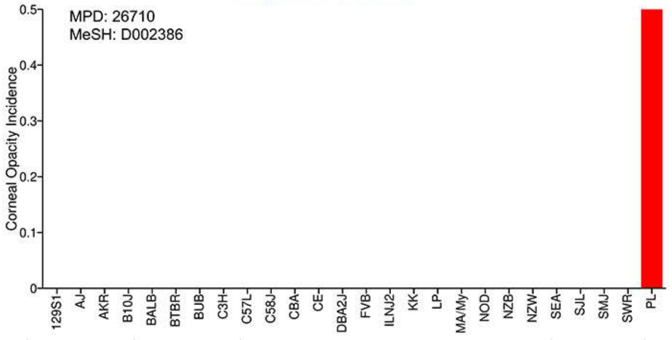


| # | Gene | SNP Flag | Haplotype | EffectSize | Pvalue | FDR | Position | LitScore | PubMed |
|---|---|---|---|---|---|---|---|---|---|
| 0 | Nid1 | 2 | | 1.000 | 0 | 0 | 13:13640077-13640077 | 0.813 | 31877171,25347398 |
| 1 | Ephx3 | 2 | | 1.000 | 0 | 0 | 17:32408705-32413853 | 0.678 | Indirect |
| 2 | Dthd1 | 2 | | 1.000 | 0 | 0 | 5:63039664-63039664 | 0.128 | Indirect |
| 3 | Mtss2 | 2 | | 1.000 | 0 | 0 | 8:111448935-111455804 | 0.476 | Indirect |
| 4 | Gabarap | 2 | | 0.425 | 0.000542049 | 0.0394737 | 11:69883098-69883526 | 0.908 | Indirect |
| 5 | Ankrd55 | 2 | | 0.182 | 0.0156415 | 0.877158 | 13:112496559-112499085 | 0.516 | Indirect |
| 6 | Hcfc1r1 | 2 | | 0.206 | 0.0414612 | 0.373036 | 17:23890264-23903432 | 0.828 | Indirect |

**Figure S3. Using the INDEL database developed here, the AI pipeline correctly identifies the causative genetic factors for agenesis of the corpus callosum (A) and cataract formation (B).** The Mouse Phenome Database (MPD) has datasets measuring the length of the corpus callosum in 16 inbred strains and the incidence of corneal opacity appearing in 26 inbred strains, which are shown in the top graph of each panel. The AI pipeline outputs the gene symbols for the genes it identifies as having the strongest genetic association with the phenotypic response pattern based upon analysis of the published literature. The output includes the calculated p-value for the genetic association, the genetic effect size, and the chromosome with the starting and ending position of each haplotype block. The color of each square within the haplotype diagram indicates the haplotype of that strain. The literature score shows the strength of the association of that gene with the measured trait, which was performed using the indicated MeSH term. Of the genes associated with agenesis of the corpus callosum, *Draxin* has a high impact INDEL, and it is the only gene with a direct association with the corpus callosum as indicated by the indicated PubMed identification number. Of the genes associated with corneal opacity, *Nid1* has a strong genetic association (genetic effect size 1.0) and is the only gene with a PubMed paper that is directly linked with the trait. *Draxin* and *Nid1* were previously shown to contain INDELs that are the murine causative genetic factors for agenesis of the corpus collosum and cataract, respectively.

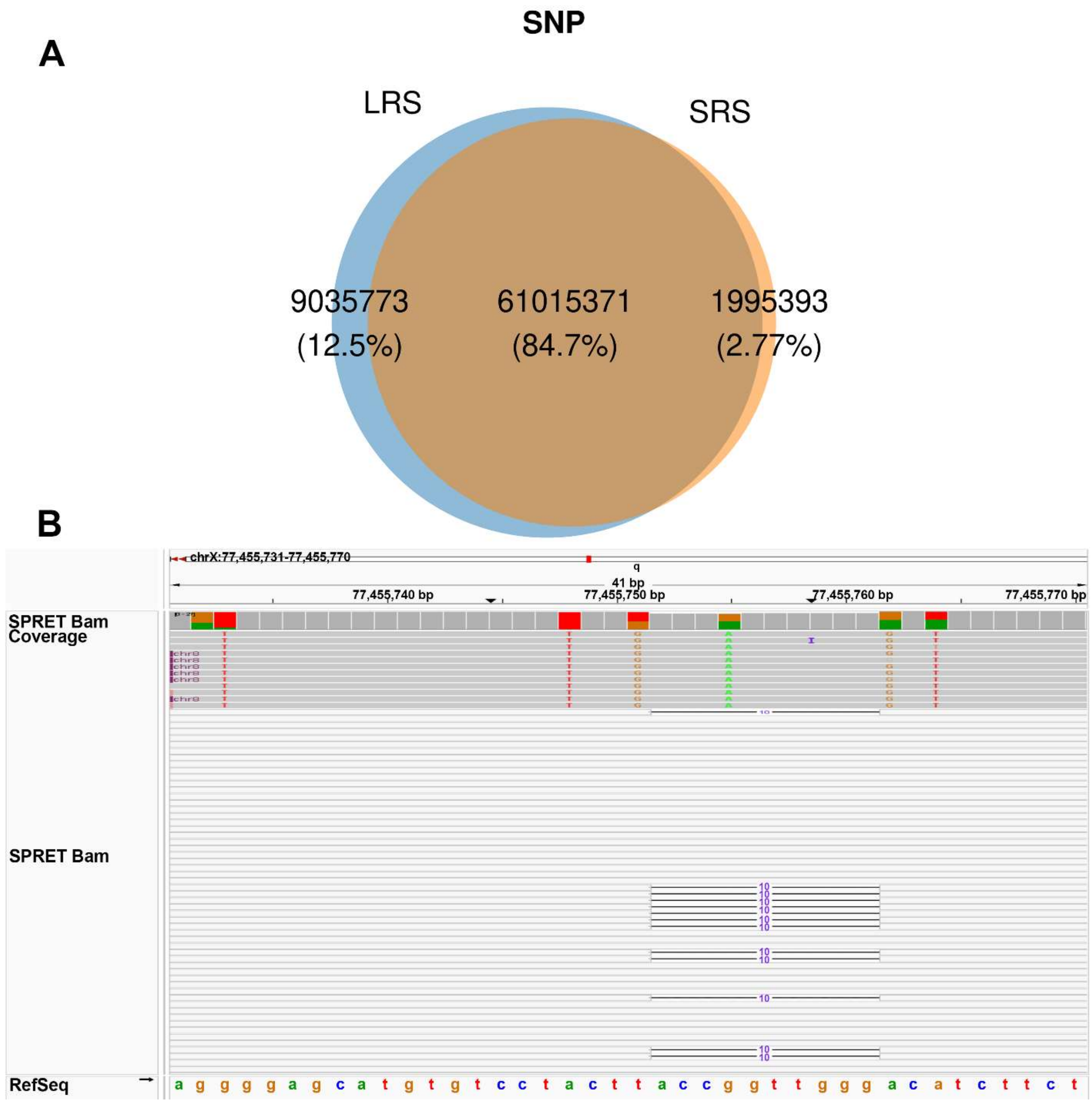


**Figure S4. Comparison of SNPs identified by LRS and SRS and visualization of a misclassified SPRET-unique SNP.** (A) A Venn diagram comparing the SNPs identified in this study (LRS), which analyzed 39 inbred strains, with those identified in a previous study using short range sequencing (SRS). The number of SNPs (and the percentages are shown in parenthesis) that are uniquely found in one dataset or are common to both datasets are shown. (B) An IGV image a SPRET-unique SNP at *chrX:77455751* whose genotype was misclassified as homozygous when it should have been labelled as heterozygous.

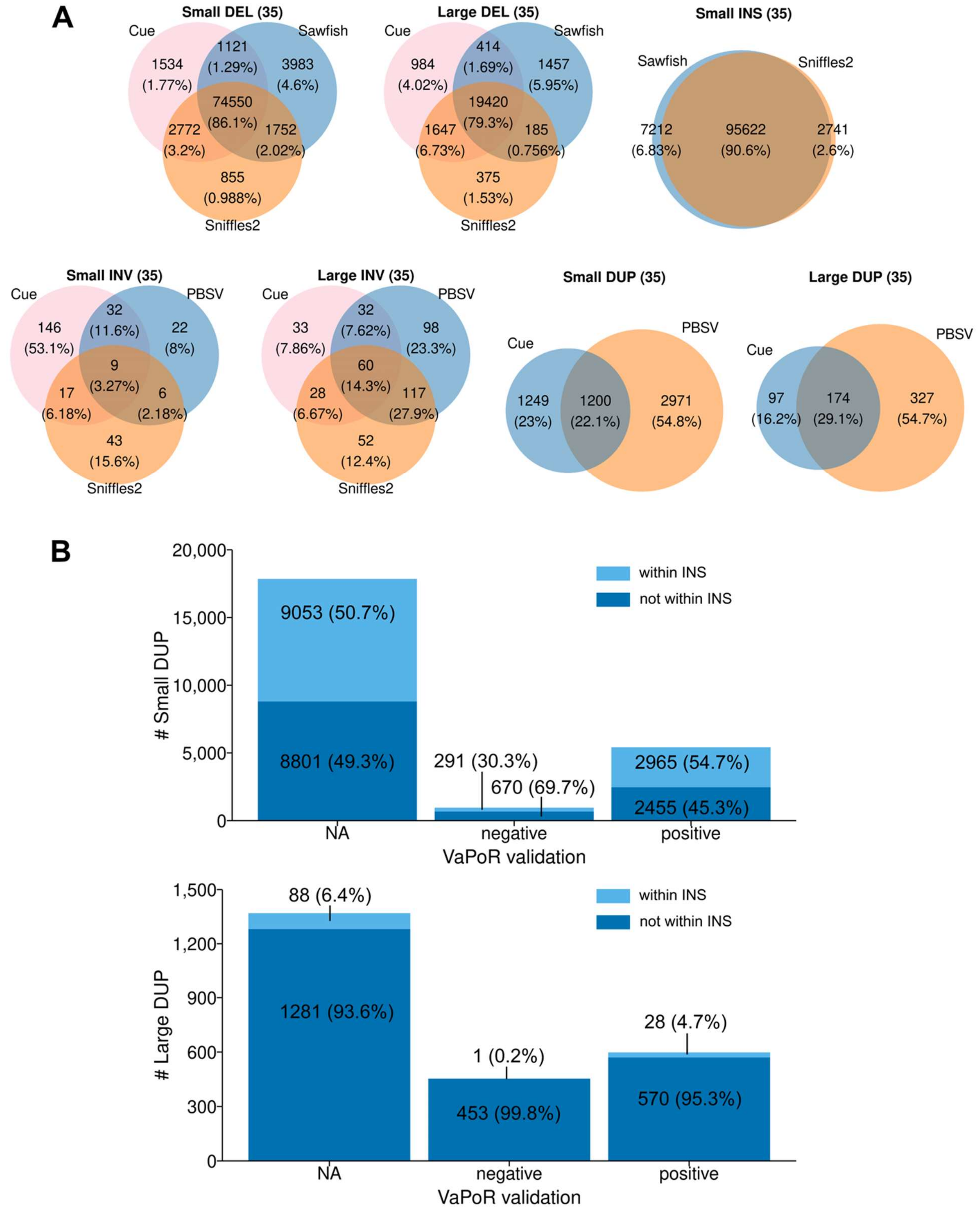
A
Small DEL (35)
Cue
Sawfish
Sniffles2
1534 (1.77%)
1121 (1.29%)
3983 (4.6%)
74550 (86.1%)
2772 (3.2%)
1752 (2.02%)
855 (0.988%)
Large DEL (35)
Cue
Sawfish
Sniffles2
984 (4.02%)
414 (1.69%)
1457 (5.95%)
19420 (79.3%)
1647 (6.73%)
185 (0.756%)
375 (1.53%)
Small INS (35)
Sawfish
Sniffles2
7212 (6.83%)
95622 (90.6%)
2741 (2.6%)
Small INV (35)
Cue
PBSV
Sniffles2
146 (53.1%)
32 (11.6%)
22 (8%)
9 (3.27%)
17 (6.18%)
6 (2.18%)
43 (15.6%)
Large INV (35)
Cue
PBSV
Sniffles2
33 (7.86%)
32 (7.62%)
98 (23.3%)
60 (14.3%)
28 (6.67%)
117 (27.9%)
52 (12.4%)
Small DUP (35)
Cue
PBSV
1249 (23%)
1200 (22.1%)
2971 (54.8%)
Large DUP (35)
Cue
PBSV
97 (16.2%)
174 (29.1%)
327 (54.7%)
B
# Small DUP
20,000
15,000
10,000
5,000
0
within INS
not within INS
9053 (50.7%)
8801 (49.3%)
291 (30.3%)
670 (69.7%)
2965 (54.7%)
2455 (45.3%)
NA
negative
positive
VaPoR validation
# Large DUP
1,500
1,200
900
600
300
0
within INS
not within INS
88 (6.4%)
1281 (93.6%)
1 (0.2%)
453 (99.8%)
28 (4.7%)
570 (95.3%)
NA
negative
positive
VaPoR validation

**Figure S5. Assessing the performance of different SV identification programs in 35 classical inbred strains.** (A) SV identification programs have an acceptable level of agreement for identification of small (50–1000 bp) and large (1 kb–500 kb) DELs, and for small INSs. The results obtained for the 35 classical inbred strains are shown in separate Venn diagrams. The number of each type of SV identified by each individual or combination of programs are shown within the circles (and percentages are shown within parenthesis). However, there was substantial divergence in the small and large INVs identified by Cue, PBSV and Sniffles2. Only the INVs that were validated by visual inspection (using the IGV) are counted. (B) Small and large DUPs identified by Cue and PBSV for the 35 inbred strains, and the VaPoR validation results are shown. Most DUPs could not be assessed (NA) by VaPoR. The assessable DUPs that passed (positive) or failed (negative) VaPoR validation are also shown. Also, 55% of the small DUPs were located within an INS, while most large DUPs were not within an INS.

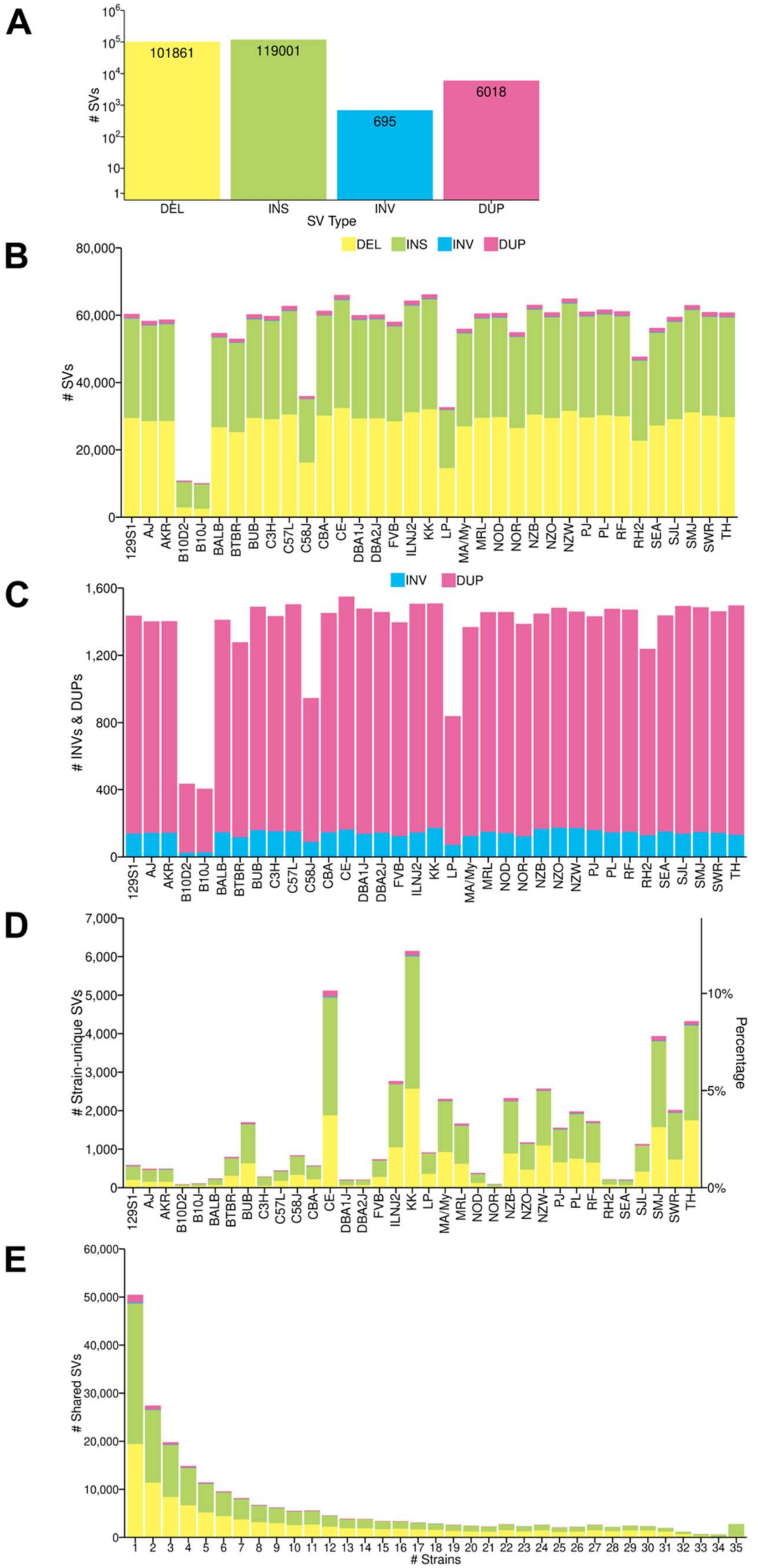

A
B
C
D
E
101861
119001
695
6018
# SVs
SV Type
DEL
INS
INV
DUP
# INVs & DUPs
# Strain-unique SVs
Percentage
# Shared SVs
# Strains

**Figure S6. Characteristics of the SV alleles among the 35 classical inbred strains.** (A) The number of different types of SVs [deletion (DEL), insertion (INS), inversion (INV) or duplication (DUP)] detected in the genome of the 35 classical inbred strains. The Y-axis is log-scale. (B) The total number of SVs detected in each strain in 35 classical inbred strains. The type of SV is indicated by the color within the bar (as shown in A). (C) The number of INV and DUP in each strain, corresponding to a magnified view of the INV and DUP categories shown in B. (D) The number (left y-axis) and percentage (right y-axis) of strain-unique SVs detected in the 35 classical inbred strains. (E) The number of strains with a shared SV allele of the indicated type are shown for the 35 classical inbred strains. Most of the minor SV alleles are shared by 1-3 strains.

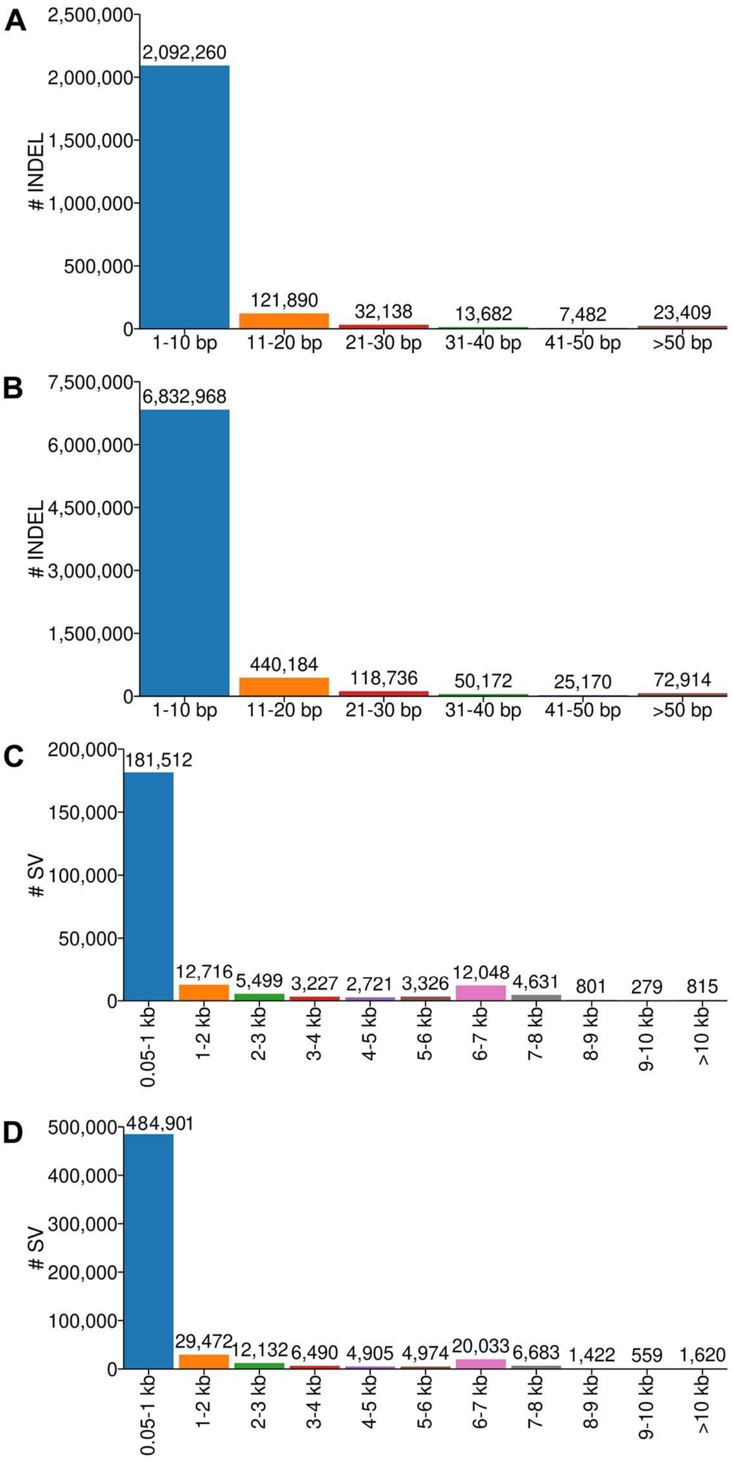


**Figure S7. Length distributions of INDELs and SVs identified in 35 classical and 39 inbred mouse strains.** (A-B) The length distribution of the INDELs identified in the 35 classical inbred strains (A) or in all 39 inbred strains (B). (C-D) The length distribution of SVs identified in the 35 classical inbred strains (C) or in all 39 inbred strains (D).

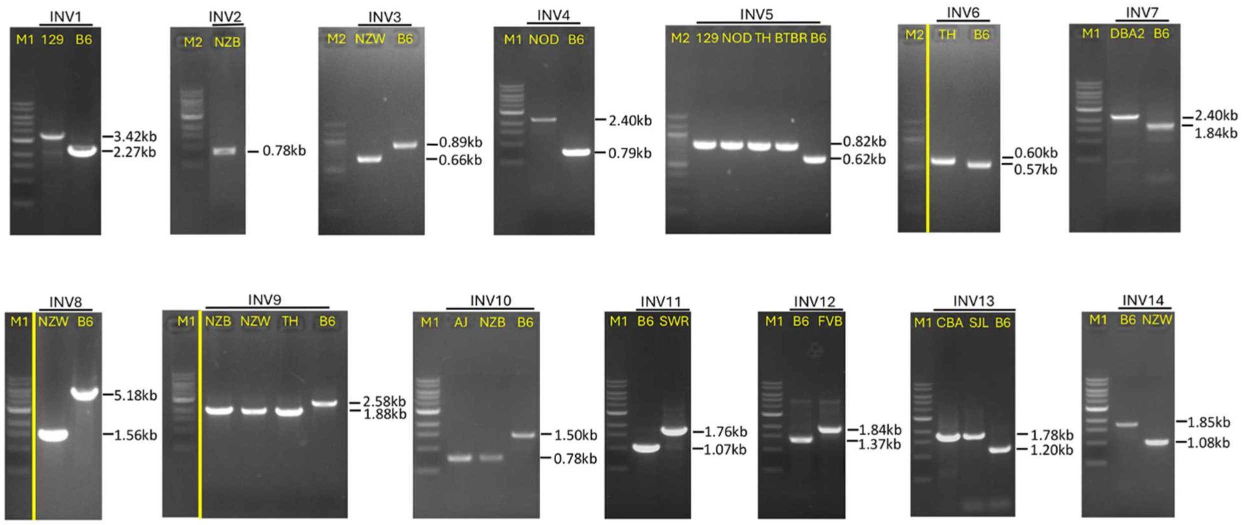


**Figure S8. Characterization of 14 INVs that were selected for experimental analysis by PCR amplification.** The PCR amplicons for each INV were resolved in 1% or 2% agarose gels. M1: 1kb ladder from New England Biolabs. M2: 100 bp ladder from New England Biolabs. All of the PCR amplicon sizes are as predicted in Table S6 based on the base-to-base-alignment of the sequences of the strain(s) with an INV and the C57BL/6J reference sequence. In the INV6, INV8 and INV9 panels the molecular weight standard lanes (M1 or M2) were juxtaposed next to the lanes with the analyzed inversions to verify the size of the amplicons shown.

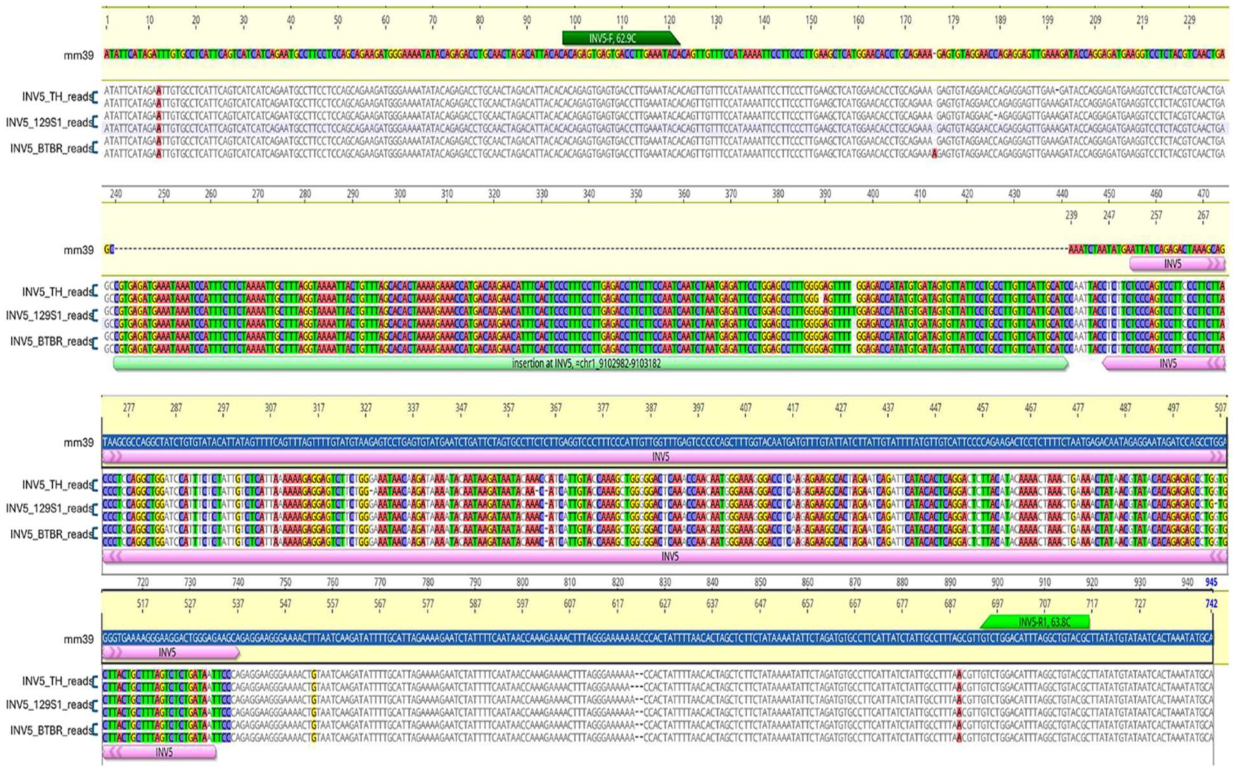


**Figure S9. Base-to-base alignment of TH, 129S1 and BTBR mice sequence (2 reads each from our LRS data) with INV5 (purple bar with arrow indicating orientations) with the corresponding location in C57BL/6J (mm39).** The template mm39 bases are highlighted (red for A, yellow for G, blue for C, green for T). For the TH, 129S1 and BTBR sequences, the gray bases are fully aligned with mm39 sequence, while the highlighted bases are mismatches and insertions (not aligned). There is a ~200bp insertion (light green bar, annotated as insertion at INV5, chr1_9102982-9103182) before INV5 sequence. The INV5-F and INV5-R1 primers used to amplify the fragments encompassing INV5 for Sanger-sequencing are indicated as short green bars. The C57BL/6J amplicon is 619 bp, while the TH, 129S1 and BTBR amplicons are predicted to be ~820 bp due to the 200 bp insertions.

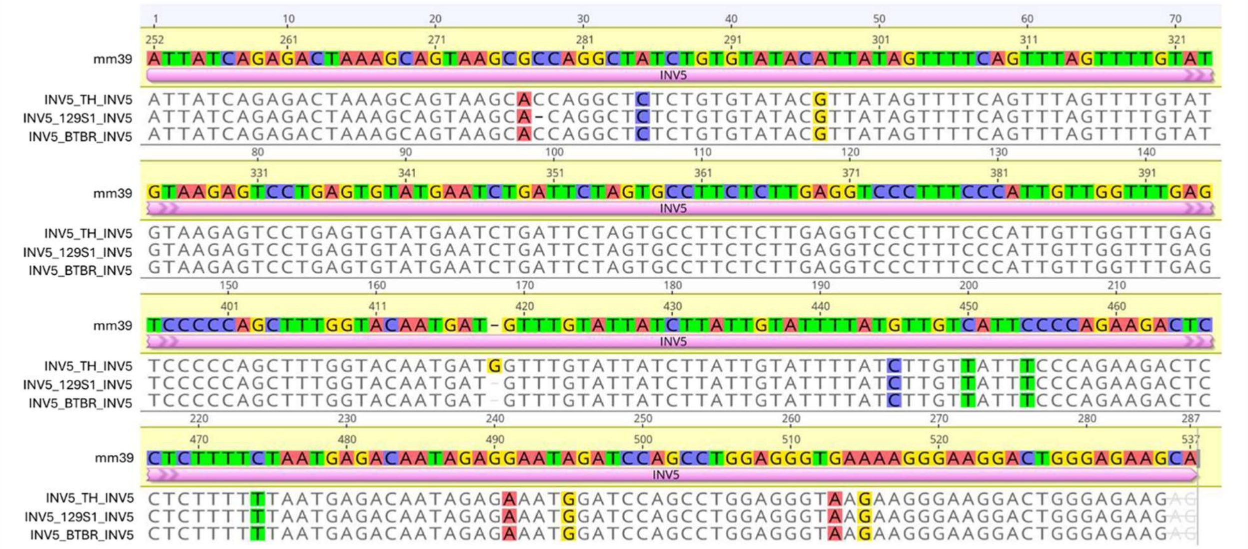

**Figure S10. Alignment of the INV5 sequences in TH, 129S1, and BTBR mice with the corresponding C57BL/6J (mm39) sequence.** The TH, 129S1 and BTBR INV5 sequences are 95% identical to that found in C57BL/6J, but were not aligned with mm39 in Figure S9 due to the presence of the inversion (opposite orientation).

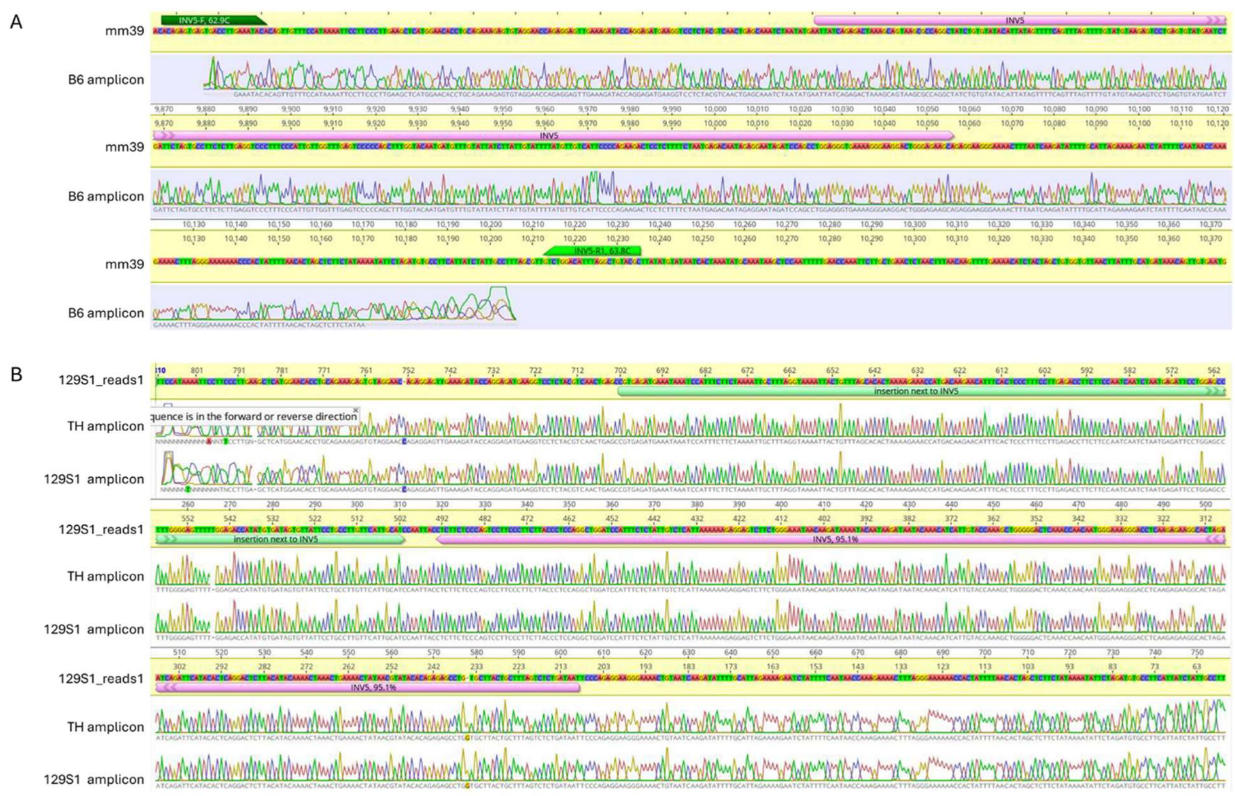


**Figure S11. Sanger sequencing of the amplicons of B6J, TH, 129S1.** INV5 amplicons shown in Figure S10 were sequenced from both primers used for PCR. Only results from one primer are shown. Panel A is the B6J amplicon aligned to mm39. Panel B is the TH and 129S1 amplicons aligned to one 129S1 read.

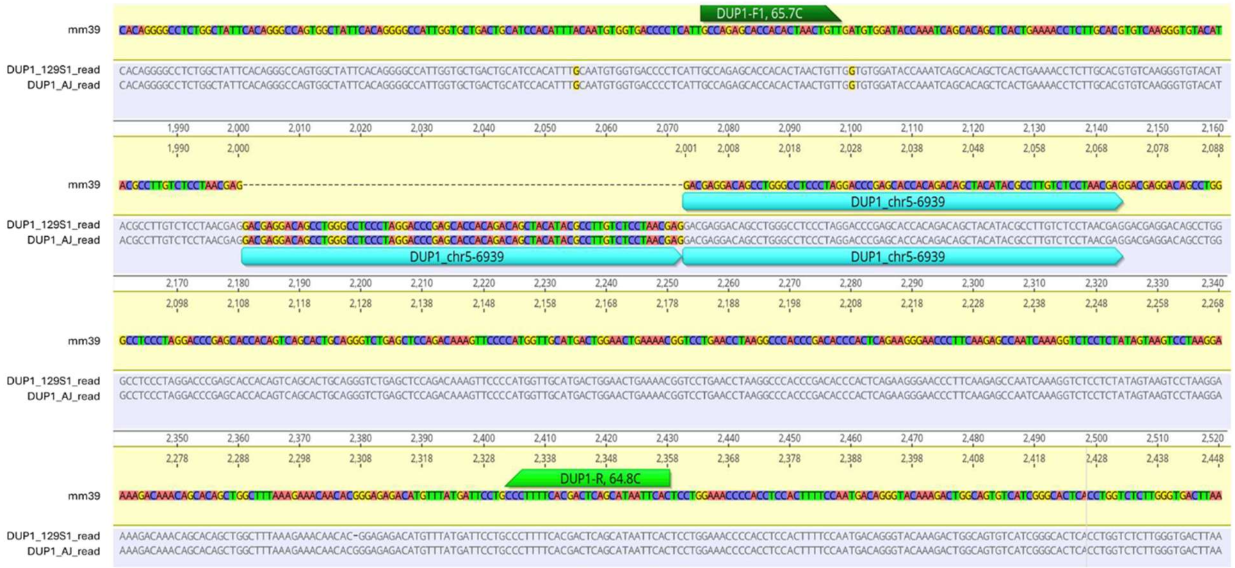


**Figure S12. Base-to-base alignment 129S1 and AJ LRS sequence where DUP1 was identified with corresponding sequence in C57BL/6J (mm39) (cyan bars).** The bases in mm39 bases are highlighted: red for A, yellow for G, blue for C, green for T. In the 129S1 and AJ sequences: gray bases are those that are fully aligned with the mm39 sequence, while highlighted bases show the mismatches and insertions that are not aligned. The predicted PCR amplicon for C57BL/6J is 509 bp, while the 129S1 and AJ amplicon are predicted to be 580 bp due to duplication of the DUP1 sequence.

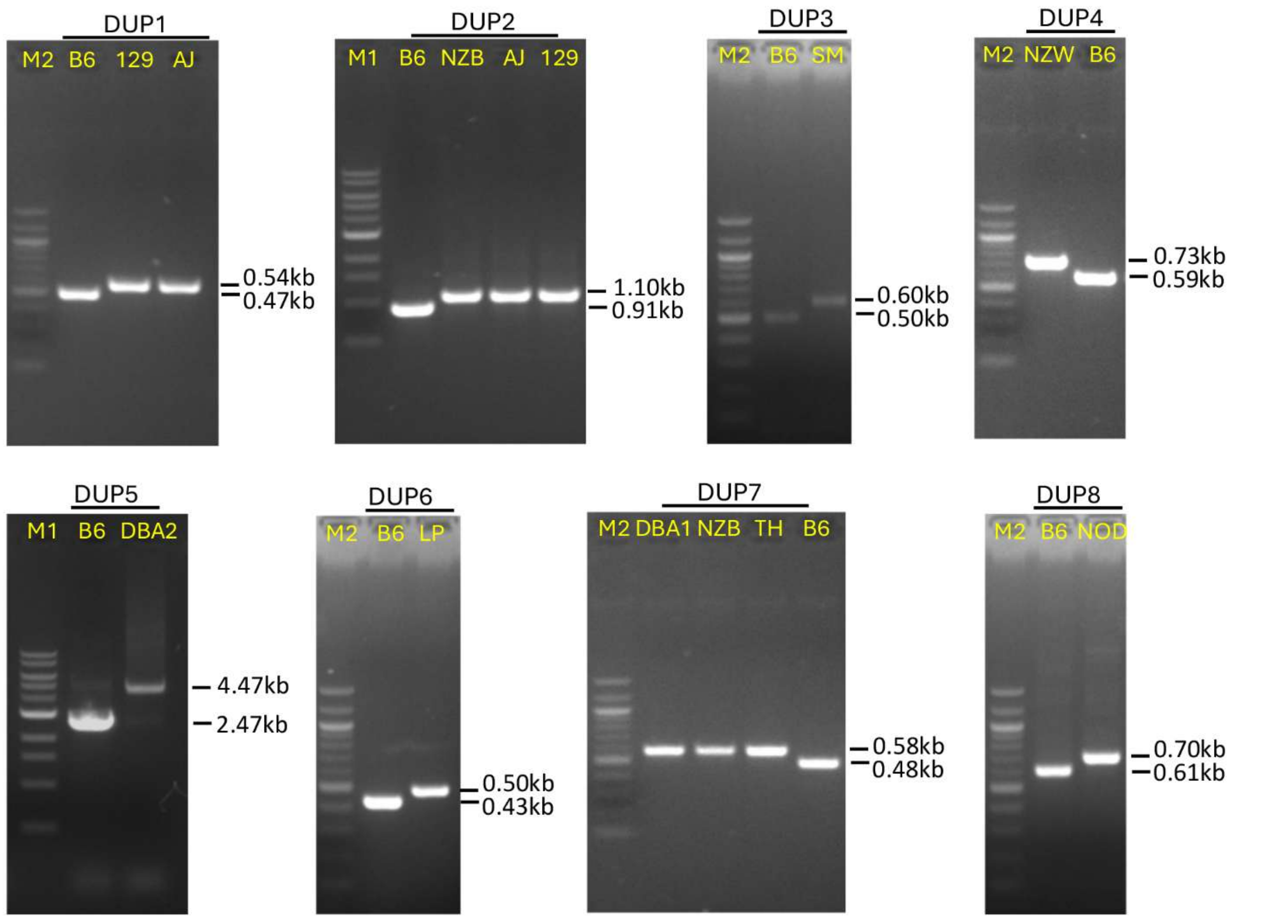


**Figure S13. PCR amplification of 8 selected DUPs.** The PCR amplicons from the indicated strains were resolved in 1% or 2% agarose gels, which were then sequenced. M1: 1kb ladder from New England Biolabs. M2: 100 bp ladder from New England Biolabs. All amplicon sizes are as predicted in Table S8 from the base-to-base-alignment of the sequences.

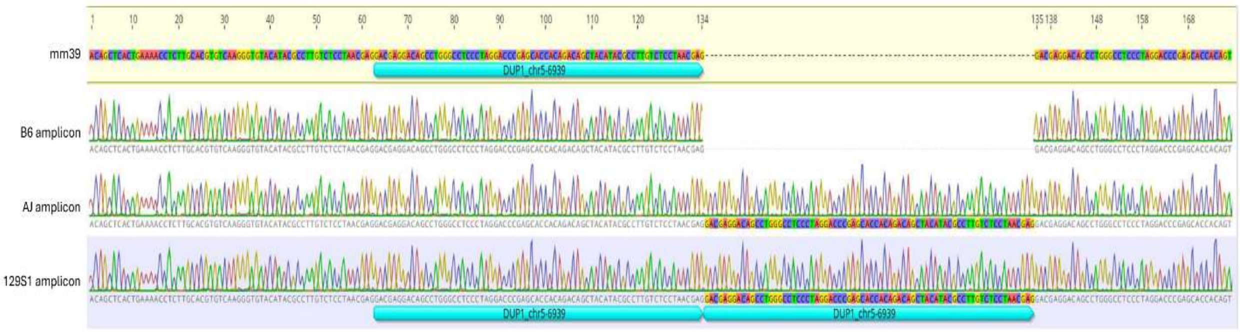


**Figure S14. Sanger sequencing of the C57BL/6J, AJ and 129S1 RT-PCR amplicons of DUP1.** The duplication present in AJ and 129S1 are readily apparent.

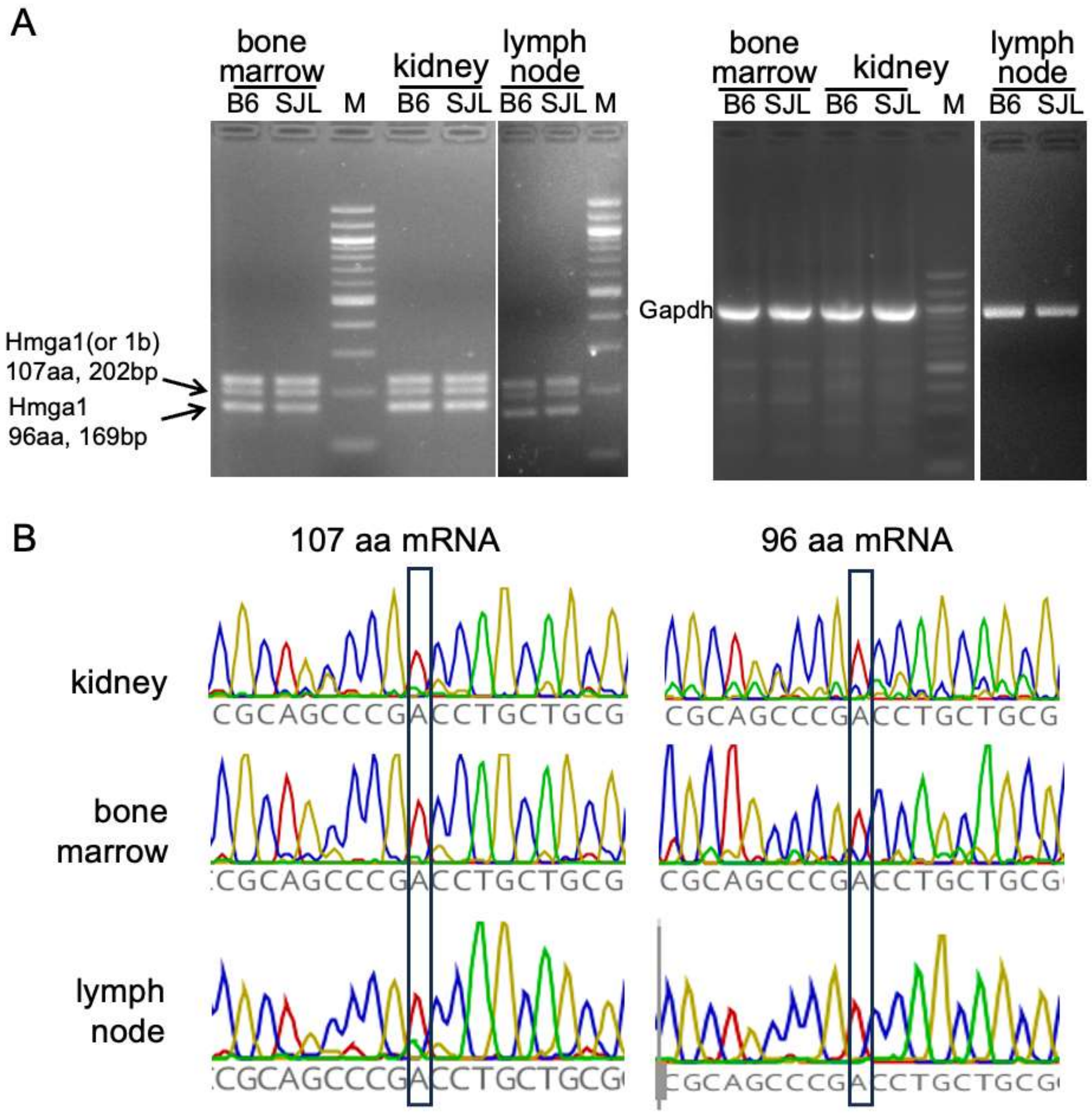


**Figure S15.** ***Hmga1* encodes the mRNAs expressed in C57BL/6J and SJL bone marrow, kidney and lymph nodes.** (A) RT-PCR was performed on hind limb bone marrow (femur, tibia), kidney and inguinal lymph node tissue obtained from C57BL/6J (B6J) and SJL mice. The amplicons encoding the *Hmga1* (96 amino acids) and *Hmga1*/*Hmga1b* 107 amino acid proteins are indicated by arrows (as in Figure 6A). The patterns of amplicon expression in kidney, bone marrow and lymph nodes (left image) are identical to that in liver and spleen obtained from B6J and SJL mice (see Figure 6B). RT-PCR of *GAPDH* amplicons are shown as a control (right image). (B) RNA was prepared from kidney, bone marrow and inguinal lymph nodes obtained from C57BL/6J mice, and RT-PCR amplicons from *Hmga1* and *Hmga1b* mRNAs were generated and sequenced. The 3' UTR of *Hmga1* mRNA of C57BL/6J mice has an A, while *Hmga1b* mRNA has a G at the boxed position (as shown in Figure 6). The sequencing results indicate that the mRNAs in C57BL/6J kidney, bone marrow and lymph nodes are encoded by *Hmga1*. This differs from the mRNAs in thymus, which are encoded by *Hmga1b* (Figure 6).

**Table S1. Characteristics of the LRS data obtained from inbred 40 strains.** The strain name, Jackson Lab #, sequence amount in gigabases (GB); and the mean, median and number of reads (in millions) for each strain are shown. The abbreviations used in the paper to shorten strain names in figures and in some of the text of the main paper are indicated. The N50 read-length distribution statistic assesses read-length quality: it represents the length of the shortest read in the group of the longest sequences that together represent >50% of the nucleotides in that sequence set. Sequencing was performed with a PacBio Revio instrument using the HiFi system.

| Strain | Abbrev | JAX Strain # | GB | # Reads (M) | Mean Read Length | Median Read Length | N50 Read length (bp) |
|---|---|---|---|---|---|---|---|
| A/J | AJ | #000646 | 85.12 | 4.69 | 18132 | 18061 | 20255 |
| C57BL/6J | B6J | #000664 | 87.65 | 5.05 | 17359 | 17127 | 18965 |
| C57BL/10J | B10J | #000665 | 92.54 | 5.76 | 16071 | 15825 | 17362 |
| C57L/J | C57L | #000668 | 92.20 | 5.92 | 15583 | 15347 | 16863 |
| CBA/J | CBA | #000656 | 88.19 | 5.12 | 17230 | 17062 | 19088 |
| DBA/1J | DBA1J | #000670 | 109.58 | 6.58 | 16644 | 16300 | 17989 |
| DBA/2J | DBA2J | #000671 | 106.62 | 6.03 | 17694 | 17341 | 19069 |
| FVB/NJ | FVB | #001800 | 97.39 | 6.07 | 16039 | 15682 | 17481 |
| KK.Cg-Ay/J | KK | #002468 | 88.64 | 5.40 | 16426 | 16085 | 17674 |
| LP/J | LP | #000676 | 89.05 | 5.49 | 16210 | 16089 | 17873 |
| NOD/ShiLtJ | NOD | #001976 | 87.21 | 4.92 | 17720 | 17496 | 19586 |
| NOR/LtJ | NOR | #002050 | 93.00 | 5.77 | 16114 | 15819 | 17722 |
| NZB/BlNJ | NZB | #000684 | 87.23 | 5.27 | 16550 | 16390 | 18197 |
| NZW/LacJ | NZW | #001058 | 90.83 | 6.10 | 14884 | 14629 | 15914 |
| TALLYHO/JngJ | TH | #005314 | 85.01 | 5.59 | 15211 | 14949 | 16694 |
| AKR/J | AKR | #000648 | 94.11 | 5.63 | 16725 | 16477 | 18181 |
| B10.D2-Hc1 H2d H2-T18c/nSnJ | B10D2 | #000463 | 98.45 | 5.89 | 16722 | 16546 | 18087 |
| C3H/HeJ | C3H | #000659 | 87.20 | 5.53 | 15754 | 15717 | 17701 |
| RF/J | RF | #000682 | 92.80 | 5.49 | 16893 | 16648 | 18797 |
| SJL/J | SJL | #000686 | 101.17 | 6.45 | 15679 | 15487 | 17057 |
| MRL/MpJ | MRL | #000486 | 96.00 | 6.17 | 15566 | 15309 | 16825 |
| NZO/HILtJ | NZO | #002105 | 88.20 | 5.03 | 17538 | 17314 | 19153 |
| WSB/EiJ | WSB | #001145 | 97.26 | 6.03 | 16135 | 15869 | 17391 |
| CAST/EiJ | CAST | #000928 | 79.22 | 5.94 | 13344 | 12413 | 15657 |
| MOLF/EiJ | MOLF | #000550 | 84.45 | 5.50 | 15345 | 14993 | 16945 |
| SPRET/EiJ | SPRET | #001146 | 82.31 | 4.86 | 16936 | 16748 | 18858 |
| SWR/J | SWR | #000689 | 85.84 | 7.04 | 12194 | 10771 | 14418 |
| 129S1/SvlmJ | 129S1 | #002448 | 84.49 | 5.16 | 16387 | 16181 | 17795 |
| BALB/cJ | BALB | #000651 | 108.88 | 6.67 | 16333 | 16056 | 18050 |
| BTBR T+ Itpr3tf/J | BTBR | #002282 | 82.31 | 4.72 | 17433 | 17224 | 18906 |
| BUB/BnJ | BUB | #000653 | 81.32 | 6.38 | 12741 | 12688 | 14612 |
| MA/MyJ | MA/My | #000677 | 87.84 | 6.22 | 14119 | 14041 | 15684 |
| C58/J | C58J | #000669 | 79.26 | 7.21 | 10997 | 10550 | 13356 |
| SM/J | SMJ | #000687 | 46.31 | 4.70 | 9864 | 9138 | 11960 |
| CE/J | CE | #000657 | 74.83 | 6.10 | 12266 | 12004 | 13752 |
| PL/J | PL | #000680 | 70.32 | 5.96 | 11803 | 11505 | 13551 |
| SEA/GnJ | SEA | #000644 | 90.93 | 7.14 | 12737 | 12637 | 14857 |
| I/LnJ | ILNJ2 | #000674 | 91.93 | 6.65 | 13829 | 13785 | 15595 |
| RHJ/LeJ | RH2 | #001591 | 81.86 | 7.67 | 10668 | 10493 | 12523 |
| P/J | PJ | #000679 | 92.88 | 5.80 | 16150 | 15778 | 17645 |
| **Total** | | | **3540.45** | **233.69** | **15301** | | |
| **Mean** | | | **88.51** | **5.84** | | | |

**Table S2. Summary of simulation study results performed to assess the effect of three distinct parameter configurations on simulation results.** The Default (D), Stringent (S) or Relaxed (R) parameters used for the parameter effect studies. For an explanation of the parameters, please refer to the GitHub page: https://github.com/acenglish/truvari/wiki/bench. In brief, the three distinct parameter configurations in Truvari varied in three criteria: refdist (the maximum allowable distance between the base call's start/end and the comparison call), pctsize (the ratio of the minimum to maximum size between the base and comparison calls), and pctovl (the ratio of overlapping bases between the two calls relative to the longest span).

| **Parameters** | **Default** | **Stringent** | **Relaxed** |
|---|---|---|---|
| **refdist** | **500** | **100** | **1000** |
| pctseq | 0.0 | 0.0 | 0.0 |
| minhaplen | 50 | 50 | 50 |
| **pctsize** | **0.7** | **0.9** | **0.5** |
| **pctovl** | **0.7** | **0.9** | **0.5** |
| typeignore | false | false | false |
| chunksize | 1000 | 1000 | 1000 |
| dup_to_ins | false | false | false |
| sizemin | 50 | 50 | 50 |
| sizefilt | 50 | 50 | 50 |
| sizemax | 100000000 | 100000000 | 100000000 |
| passonly | true | true | true |

**Table S3. The results of simulations assessing the ability of Cue, Sniffles2, Sawfish, PBSV, and Dipcall to identify the indicated types of SVs using the D, S, and R parameters.** For each simulation, 1000 SVs of a single type (DEL, INS, INV, DUP) within a defined size [small 50–1000 bp; or large 1–100 kb] were artificially inserted at random positions into the GRCm39 reference sequence. Truvari was used to evaluate precision (P), recall (R), and F1 statistic for different SV types/sizes using the three D, S, R parameter configurations, which were calculated based on the ability of each program to detect the inserted SVs. Cue, Sniffles2, Sawfish, and PBSV analyzed HiFi long-read sequencing data (30× coverage) that was aligned using minimap2. The simulations were performed using PBSIM3. The genomic sequences analyzed by Dipcall were assembled using HiFiasm. NA: not assessed. W* and $W^{\$}$: withdrawn for the reasons discussed in the manuscript. Only small differences were obtained with different parameters (which were mostly seen for small DUPs). These results indicates that parameter differences would not alter our choice of SV calling program.

| | | Cue | | | Sniffles2 | | | Sawfish | | | PBSV | | | Dipcall | | |
|---|---|---|---|---|---|---|---|---|---|---|---|---|---|---|---|---|
| | | D | S | R | D | S | R | D | S | R | D | S | R | D | S | R |
| Small DEL | P | 1 | 1 | 1 | 1 | 1 | 1 | 1 | 1 | 1 | 1 | 1 | 1 | 0.99 | 0.99 | 0.99 |
| | R | 0.994 | 0.994 | 0.994 | 0.999 | 0.999 | 0.999 | 1 | 1 | 1 | 1 | 1 | 1 | 0.702 | 0.702 | 0.702 |
| | F1 | 0.996 | 0.996 | 0.996 | 0.9995 | 0.9995 | 0.9995 | 1 | 1 | 1 | 1 | 1 | 1 | 0.821 | 0.821 | 0.821 |
| Large DEL | P | 1 | 1 | 1 | 0.991 | 0.991 | 0.991 | 0.989 | 0.989 | 0.989 | 0.997 | 0.997 | 0.997 | 1 | 1 | 1 |
| | R | 0.998 | 0.998 | 0.998 | 0.548 | 0.548 | 0.548 | 0.995 | 0.995 | 0.995 | 0.648 | 0.648 | 0.648 | 0.494 | 0.494 | 0.494 |
| | F1 | 0.999 | 0.999 | 0.999 | 0.706 | 0.706 | 0.706 | 0.992 | 0.992 | 0.992 | 0.785 | 0.785 | 0.785 | 0.661 | 0.661 | 0.661 |
| Small INS | P | NA | | | 0.998 | 0.998 | 0.998 | 1 | 1 | 1 | 1 | 1 | 1 | 0.987 | 0.985 | 0.987 |
| | R | NA | | | 0.978 | 0.978 | 0.978 | 0.982 | 0.982 | 0.982 | 0.979 | 0.979 | 0.979 | 0.811 | 0.81 | 0.811 |
| | F1 | NA | | | 0.988 | 0.988 | 0.988 | 0.991 | 0.991 | 0.991 | 0.989 | 0.989 | 0.989 | 0.89 | 0.889 | 0.89 |
| Large INS | P | NA | | | 1 | 1 | 1 | 1 | 1 | 1 | 1 | 1 | 1 | 1 | 1 | 1 |
| | R | NA | | | 0.182 | 0.182 | 0.182 | 0.358 | 0.358 | 0.358 | 0.13 | 0.13 | 0.13 | 0.936 | 0.936 | 0.936 |
| | F1 | NA | | | 0.308 | 0.308 | 0.308 | 0.527 | 0.527 | 0.527 | 0.23 | 0.23 | 0.23 | 0.967 | 0.967 | 0.967 |
| Small INV | P | 0.999 | 0.974 | 1.0 | 0.908 | 0.906 | 0.929 | W* | | | 0.967 | 0.967 | 0.967 | NA | | |
| | R | 0.909 | 0.886 | 0.91 | 0.434 | 0.433 | 0.444 | W* | | | 0.758 | 0.758 | 0.758 | NA | | |
| | F1 | 0.952 | 0.928 | 0953 | 0.587 | 0.586 | 0.601 | W* | | | 0.85 | 0.85 | 0.85 | NA | | |
| Large INV | P | 0.999 | 0.999 | 0.999 | 0.994 | 0.994 | 0.994 | W* | | | 0.994 | 0.994 | 0.994 | NA | | |
| | R | 0.999 | 0.999 | 0.999 | 0.998 | 0.998 | 0.998 | W* | | | 0.901 | 0.901 | 0.901 | NA | | |
| | F1 | 0.999 | 0.999 | 0.999 | 0.996 | 0.996 | 0.996 | W* | | | 0.9454 | 0.9454 | 0.9454 | NA | | |
| Small DUP | P | 0.997 | 0.942 | 1.0 | 0 | | | $W^{\$}$ | | | 0.792 | 0.777 | 0.792 | NA | | |
| | R | 0.976 | 0.921 | 0.978 | 0 | | | $W^{\$}$ | | | 0.795 | 0.78 | 0.795 | NA | | |
| | F1 | 0.987 | 0.931 | 0.989 | 0 | | | $W^{\$}$ | | | 0.793 | 0.778 | 0.793 | NA | | |
| Large DUP | P | 0.999 | 0.999 | 0.999 | 0.92 | 0.92 | 0.934 | 0.956 | 0.956 | 0.96 | 0.986 | 0.984 | 0.986 | NA | | |
| | R | 0.993 | 0.993 | 0.993 | 0.916 | 0.916 | 0.93 | 0.762 | 0.762 | 0.765 | 0.982 | 0.98 | 0.982 | NA | | |
| | F1 | 0.996 | 0.996 | 0.996 | 0.918 | 0.918 | 0.932 | 0.848 | 0.848 | 0.851 | 0.984 | 0.982 | 0.984 | NA | | |

**Table S4. The functional consequences of SVs that were predicted by VEP.** Their estimated severity (HIGH, MODERATE, LOW, MODIFIER) and the type of effect (consequences) was determined for SVs in all 39 strains or those in the 35 classical inbred strains.

| Impact | Consequences | #SV 39 strains | #SV 35 strains |
|---|---|---|---|
| HIGH | transcript_ablation | 2068 | 1079 |
| | splice_acceptor_variant | 33 | 14 |
| | splice_donor_variant | 44 | 24 |
| | stop_gained | 29 | 10 |
| | frameshift_variant | 102 | 55 |
| | stop_lost | 171 | 92 |
| | start_lost | 4 | 1 |
| | transcript_amplification | 191 | 130 |
| | feature_elongation | 5823 | 2538 |
| | feature_truncation | 5555 | 2388 |
| MODERATE | inframe_insertion | 39 | 24 |
| | inframe_deletion | 183 | 88 |
| | protein_altering_variant | 5 | 2 |
| LOW | splice_donor_5th_base_variant | 44 | 24 |
| | splice_region_variant | 19 | 9 |
| | splice_donor_region_variant | 7 | 3 |
| | splice_polypyrimidine_tract_variant | 275 | 98 |
| | start_retained_variant | 4 | 1 |
| | stop_retained_variant | 19 | 12 |
| MODIFIER | coding_sequence_variant | 2389 | 1398 |
| | mature_miRNA_variant | 1 | 0 |
| | 5_prime_UTR_variant | 1171 | 636 |
| | 3_prime_UTR_variant | 4100 | 1808 |
| | non_coding_transcript_exon_variant | 7140 | 3161 |
| | intron_variant | 244001 | 97134 |
| | non_coding_transcript_variant | 41051 | 17103 |
| | coding_transcript_variant | 108 | 68 |
| | upstream_gene_variant | 11946 | 4955 |
| | downstream_gene_variant | 14283 | 5984 |
| | regulatory_region_ablation | 7130 | 3537 |
| | regulatory_region_amplification | 358 | 253 |
| | regulatory_region_variant | 66411 | 29290 |
| | intergenic_variant | 290358 | 117312 |

**Table S5. The number of insertion and deletion SVs identified by VEP analysis as having a high impact (protein coding) in all 39 strains or in the 35 classical inbred strains are shown.**

| | 39 strains | | 35 strains | |
|---|---|---|---|---|
| | # SV | # Unique Genes | # SV | # Unique Genes |
| Small INS | 2082 | 1899 | 801 | 765 |
| Large INS | 822 | 740 | 518 | 487 |
| Small DEL | 1688 | 1535 | 705 | 654 |
| Large DEL | 590 | 536 | 293 | 270 |

**Table S6**. **Summary of the 14 INVs that were further characterized.** The table shows the number for each INV; the chromosome, starting and ending position, and the length of the INV; and lists the strain(s) with the INV. Each inversion was characterized by examining base-to-base alignments with the reference sequence (LRS Aligned). This table also shows the expected size of PCR amplicons generated from C57BL/6J and from strains with the INV, and whether an inversion was assessed by PCR or DNA sequence analysis.

| INV # | Chr | Start | End | Size (bp) | Strains | Amplicons Size (kb) | | LRS Aligned | PCR Valid | DNA Seq |
|---|---|---|---|---|---|---|---|---|---|---|
| | | | | | | B6J | Other strain | | | |
| 1 | 4 | 93274454 | 93274794 | 341 | 129S1, C57L, RH | 2.18 | 3.36, 129S1 | yes | yes | yes |
| 2 | 4 | 101587861 | 101588296 | 436 | NZB, NZO | 2.76 | 0.78, NZB | yes | yes | yes |
| 3 | 12 | 25652919 | 25653278 | 360 | C57L, NZW | 0.89 | 0.66, NZW | yes | yes | yes |
| 4 | 11 | 65438162 | 65438482 | 321 | CE, LP, NOD, SWR | 0.79 | 2.4kb, NOD | yes | yes | yes |
| 5 | 1 | 9101845 | 9102130 | 286 | 129S1, AKR, BTBR, C57L, ILNJ, LP, NOD, NOR, PJ, PL, SMJ, SWR, TH | 0.62 | 0.82, TH, 129S1, BTBR, NOD | yes | yes | yes |
| 6 | 2 | 42438092 | 42438405 | 313 | TH | 0.57 | 0.60, TH | yes | yes | yes |
| 7 | 2 | 65233901 | 65235066 | 1165 | DBA2J, NOD | 1.65 | 2.39, DBA2J | yes | yes | yes |
| 8 | 12 | 107286406 | 107288893 | 2487 | BUB, MA/My, NZW, PJ, SEA | 5.18 | 1.56, NZW, Ma/My | yes | yes | yes |
| 9 | 7 | 54520508 | 54522578 | 2070 | ILNJ, KK, MA/My, NZB, NZW, SJL, TH | 2.58 | 1.88, NZB, NZW, TH | yes | yes | yes |
| 10 | 5 | 148050415 | 148050807 | 393 | AJ, AKR, BALB, C3H, NOD, NOR, NZB | 1.50 | 0.78, AJ, NZB | yes | yes | yes |
| 11 | 13 | 58062240 | 58062869 | 630 | SWR | 1.76 | 1.07, SWR | yes | yes | yes |
| 12 | 14 | 104744106 | 104744629 | 524 | FVB | 1.37 | 1.84, FVB | yes | yes | yes |
| 13 | 2 | 155899201 | 155899509 | 309 | 129S1, BALB, C3H, C57L, CBA, FVB, NOD, NZO, NZW, SJL, SWR, TH | 1.2 | 1.78, CBA, SJL | yes | yes | yes |
| 14 | 4 | 15993614 | 15994386 | 773 | NZW | 1.08 | 1.85, NZW | yes | yes | yes |

**Table S7. PCR and sequencing primers used for validation of INVs.**

| Name | Sequence | Name | Sequence |
|---|---|---|---|
| INV1-F2 | AAGACAGCACTTAAATGGGCCTG | INV13-F | CTGAGAATCACTGGGAAAGAGTGTA |
| INV1-R3 | GGAATGTTATAGCAGCCTTGGAAGAC | INV13-R | CAAATAAACCCTTCCCTGCTGTCCA |
| INV2-F1 | TGAGACTGACAATAATATCATATGGGTAACA | INV14-F | AGAGAAGTAGATATCAACTCCAGCTGT |
| INV2-R | AATGGTCTGTTTAGTAAATGGCACTGG | INV14-R | CTTTAAGTGACGCTTATGGTAGTGGA |
| INV3-F | CCAGTGAGAAGGCAGTTTCTTCT | Below are primers used for sequencing only | |
| INV3-R1 | CATCTCTTTTAGAAAAATCCTCTCCTTTC | INV1-seq1 | GCAGAGAAGCACCTGAAAAAATGC |
| INV4-F | GCTTTGAATTTATATTTAGAGCCTCCAGG | INV1-seq3 | TTAGCTCCTTGGGTACTTTCTCTAG |
| INV4-R | CTGTGGGCGAAAATAAACCATACTC | INV1-seq4 | AGCTTGGTCAGAGGAGTGGA |
| INV5-F | ACAGAGTGAGTGACCTTGAAATACA | INV1-seq5 | GTGTCCAATGGGCCTCTCTTTC |
| INV5-R1 | GCGTACAGCCTAAATGTCCAGAC | INV1-F | AGTTGTAGATCCAGTGAAAGAGTAAGAG |
| INV6-F | CAGGGTTCAGCTCCTATCACTCACA | INV1-R1 | CTCCTCTCAAGGCCTGGTTAAAGAA |
| INV6-R | GCAATGAAATGCTTTAACACGCACTC | INV4-seq2 | CCTGGTCGAAGAGGATCCATAC |
| INV7-F | AGTGGCTATTTCTTTCCAGCACAGTAG | INV4-seq3 | CCTACCATAAGAACAGTACATTCACG |
| INV7-R1 | CGTGGGAGTCAGTCATGTTACACTC | INV4-seqR | GAAAGAGAGGCCCATTGGACAC |
| INV8-F1 | TTGCAATAAAGACAGTGACAGTCATGC | INV4-seq2R | GTATGGATCCTCTTCGACCAGG |
| INV8-R | GGACAATGGGTTATACTGAGAACACC | INV7-seq1 | GACAAGTTTCAGGTCTAGCAGATAT |
| INV9-F1 | GCAGACAGACCATCAAAATACAGTGC | INV7-seq2 | CTTTGAATTTTCCTATGAGCACAACATTC |
| INV9-R1 | GGAATTTCATTGTTTATGGACACAGGAG | INV7-F1 | ACTGATTAGTTGACTCAAGGATGAGG |
| INV10-F | TGTTTCATAGTTTTGATATAAATGTCTGGCT | INV8-seq1 | CCAGTGGGTGGCTTTGAATAAG |
| INV10-R | TCCCATGTAGGAATGTGACTGAACAT | INV8-seq2 | GGATCAATTTGCATTCTTCTACATACTG |
| INV11-F | GAACAAACTCCACAAGCATGCCA | INV8-seq3 | GGCCACTGATTTGTTTGAGTTAACT |
| INV11-R | GTAACTTTGTAGCTGATGGTCTAATAGGT | INV8-seq4 | TGTCTTCCTCCACTGATTAAGTTAGG |
| INV12-F | TGGTACAACACTAATTTAGAATGCTT | INV9-seq1 | CAAGAAACAAAAATCGCTCCAATAAAGA |
| INV12-R | AAGGGAAGTCTGGTCCAAGTGT | INV9-seq2 | GTGTGGTTCTCGTATGTGTATATGT |

**Table S8. Summary of 8 DUPs that were further characterized.** The table shows the number of each DUP; the chromosome, starting and ending position, and the length of the DUP; and it lists the strain(s) with each DUP. The DUPs were characterized by examining base-to-base alignments with the C57BL/6J reference sequence (LRS Aligned). This table also shows the predicted size of PCR amplicons generated from C57BL/6J and from strains with a DUP, and whether a DUP was assessed by PCR or DNA sequence analysis.

| DUP # | Chr | Start | End | Size (bp) | Strains | Amplicons Size (kb) | | LRS Aligned | PCR Valid | DNA Seq |
|---|---|---|---|---|---|---|---|---|---|---|
| | | | | | | B6J | Other strain | | | |
| 1 | 5 | 117236939 | 117237010 | 72 | 129S1, AJ, AKR, BALB, BUB, C57L, MRL, PJ, PL, RH2, SEA, SJL, SMJ | 0.51 | 0.58, 129S1, AJ | yes | yes | yes |
| 2 | 9 | 106607630 | 106607824 | 194 | 129S1, AJ, AKR, BALB, BUB, C3H, C57L, CE, FVB, ILNJ2, MA/My, MRL, NOD, NOR, NZB, NZO, NZW, PL, RF, RH2, SJL, SMJ, SWR, TH | 0.91 | 1.10, NZB, AJ, 129S1 | yes | yes | yes |
| 3 | 2 | 72389468 | 72389569 | 102 | PJ, SMJ | 0.50 | 0.60, SMJ | yes | yes | yes |
| 4 | 10 | 24614233 | 24614362 | 129 | NZO, NZW | 0.59 | 0.73, NZW | yes | yes | yes |
| 5 | 6 | 94865599 | 94867710 | 2111 | DBA2J | 2.47 | 4.47, DBA2J | yes | yes | yes |
| 6 | 15 | 7878156 | 7878216 | 60 | LP | 0.43 | 0.50, LP | yes | yes | yes |
| 7 | 12 | 29671776 | 29671839 | 64 | BTBR, C3H, C57L, CBA, DBA1J, KK, MRL, NZB, NZO, TH | 0.48 | 0.58, NZB, DBA1J, TH | yes | yes | yes |
| 8 | 8 | 78781191 | 78781284 | 93 | NOD, NOR | 0.61 | 0.70, NOD | yes | yes | yes |

**Table S9. PCR and sequencing primers used for validation of DUPs.**

| Name | Sequence |
|---|---|
| DUP1-F | ATTGGTGCTGACTGCATCCACATT |
| DUP1-R | GTGAATTATGCTGAGTCGTGAAAAGGG |
| DUP2-F | CTCCTAGCCTTTAATGGTGACCTCA |
| DUP2-R | CTCCTAGTGTTGACAGTCTAGCTCC |
| DUP3-F | GTGATCCAGGTTTTCTTTGAAGGTCTC |
| DUP3-R | GAGATGAAGAAACAACTGGGACCAG |
| DUP4-F | CAAGTTCAGTGAGAGACCTCTGTAAGA |
| DUP4-R | TGCTGCTTTCCATCCAAGTCACC |
| DUP5-F | CTTAAGGGTAAAAGTCAGCAGTACATGA |
| DUP5-R | GAAGATTTGTAATTTGCCCTGAAATTCAGTT |
| DUP5-R1 | GGTATGCCCCTAAATGTCTTTTATAACAATC |
| DUP6-F | AGGTTAACATCATCATTGTCCTCACTTT |
| DUP6-R | CTTCCTCTCATCTTTGCCACCATGT |
| DUP7-F | GAAGGATATATTTTCACTTTGCTCTGAGG |
| DUP7-R | GCTTCAGAATGCAGCCATAGC |
| DUP8-F | TCCATCTTCTAAATTCCAATAATCCCACC |
| DUP8-R | AATTACTTCAATCAGTGGTATCCGAGAT |
| DUP5-s1 | GTGACTGGCTGACTACATAGAGACA |
| DUP5-s2 | TGAAGGAGTGAAGCCGCGAAG |
| DUP5-s3 | CCATAGTACCCAAATATTGATTG |

**Table S10. Summary of the SNPs, INDELs, and SVs identified when our C57BL/6J LRS data was compared with the GRCm39 reference sequence for C57BL/6J.**

| Variant Type | Subtype | Count |
|---|---|---|
| SNP | - | 11751 |
| INDEL | - | 9851 |
| Deletion | Small DEL | 603 |
| | Large DEL | 114 |
| | **Total DEL** | **717** |
| Insertion | Small INS | 4040 |
| | Large INS | 228 |
| | **Total INS** | **4268** |
| Inversion | Small INV | 5 |
| | Large INV | 9 |
| | **Total INV** | **14** |
| Duplication | Small DUP | 269 |
| | Large DUP | 23 |
| | **Total DUP** | **292** |
